# Biological Control of Pest Non-Marine Molluscs: A Pacific Perspective on Risks to Non-Target Organisms

**DOI:** 10.3390/insects12070583

**Published:** 2021-06-28

**Authors:** Carl C. Christensen, Robert H. Cowie, Norine W. Yeung, Kenneth A. Hayes

**Affiliations:** 1 Bernice Pauahi Bishop Museum, Honolulu, HI 96822, USA; carl.christensen@bishopmuseum.org (C.C.C.); cowie@hawaii.edu (R.H.C.); nyeung@hawaii.edu (N.W.Y.); 2 Pacific Biosciences Research Center, University of Hawaii, Honolulu, HI 96822, USA

**Keywords:** biological control, non-target impacts, snails, slugs, *Euglandina*, *Platydemus*, Sciomyzidae, Sarcophagidae, *Phasmarhabditis*

## Abstract

**Simple Summary:**

As malacologists long concerned with conservation of molluscs, we present empirical evidence supporting the proposition that biological control of nonmarine mollusc pests has generally not been demonstrated to be safe and effective, which are the basic measures of success. Yet claims of success often accompany contemporary biological control programs, although without rigorous evaluations. Failed molluscan biocontrol programs include well known classical control efforts that continue to devastate native biodiversity, especially on Pacific islands, as well as more contemporary programs that claim to be safer, with minimal non-target impacts. We do not condemn all biological control programs as ineffective, unsafe, and poorly evaluated, but emphasize the need for programs targeting non-marine molluscs to incorporate the lessons learned from past failures, and to do a better job of defining and measuring success both pre- and post-release of biocontrol agents. Most importantly, we call for the biocontrol community not to rely on entomologists with backgrounds in use of host-specific agents, who yet promote generalist parasites/predators for mollusc control, but to engage more actively with those knowledgeable in molluscan biology, particularly conservation. In doing so, maybe some programs targeting molluscan pests can become safe and effective.

**Abstract:**

Classic biological control of pest non-marine molluscs has a long history of disastrous outcomes, and despite claims to the contrary, few advances have been made to ensure that contemporary biocontrol efforts targeting molluscs are safe and effective. For more than half a century, malacologists have warned of the dangers in applying practices developed in the field of insect biological control, where biocontrol agents are often highly host-specific, to the use of generalist predators and parasites against non-marine mollusc pests. Unfortunately, many of the lessons that should have been learned from these failed biocontrol programs have not been rigorously applied to contemporary efforts. Here, we briefly review the failures of past non-marine mollusc biocontrol efforts in the Pacific islands and their adverse environmental impacts that continue to reverberate across ecosystems. We highlight the fact that none of these past programs has ever been demonstrated to be effective against targeted species, and at least two (the snails *Euglandina* spp. and the flatworm *Platydemus manokwari*) are implicated in the extinction of hundreds of snail species endemic to Pacific islands. We also highlight other recent efforts, including the proposed use of sarcophagid flies and nematodes in the genus *Phasmarhabditis*, that clearly illustrate the false claims that past bad practices are not being repeated. We are not making the claim that biocontrol programs can never be safe and effective. Instead, we hope that in highlighting the need for robust controls, clear and measurable definitions of success, and a broader understanding of ecosystem level interactions within a rigorous scientific framework are all necessary before claims of success can be made by biocontrol advocates. Without such amendments to contemporary biocontrol programs, it will be impossible to avoid repeating the failures of non-marine mollusc biocontrol programs to date.

## 1. Introduction

Biological control has often been praised as an efficacious and environmentally benign method of controlling pest animal species. We do not outright dismiss the possibility, in specific cases where appropriate studies pre- and post-release have been done, that biological control can be safe and effective, especially in cases where narrow target-specific agents have been rigorously identified, studied and used to control plant and arthropod pests. For example, successful Hawaiian programs without known adverse environmental impacts include control of the spiraling whitefly, *Aleurodiscus dispersus* [1], and the erythrina gall wasp, *Quadrastichus erythrinae* [2]. That is not the case, however, when generalist predators or non-host specific parasites are employed, as non-target species may be adversely impacted [3,4,5,6]. This is especially so with historical biocontrol programs targeting non-marine molluscs. As one authority has noted, “the history of biological control of mollusc pests provides one of the so-called ‘best’ examples of how poorly planned biological control can itself result in the extinction of non-target species” [7] (p. 133). Accordingly, any proposal for the use of biological control agents targeting pest species of non-marine molluscs must be evaluated in the light of lessons learned from that long and unhappy history, as well as a modern understanding of the ecological and evolutionary complexity of species interactions. Our view of control programs targeting pest non-marine molluscs is shaped by studies of the systematics and conservation of the non-marine molluscs that have been the collateral damage of those programs, particularly on Pacific islands (e.g., [8,9,10,11,12,13,14,15,16,17,18,19,20]).

Biocontrol of non-marine molluscs has a long history fraught with unintended consequences. The earliest such effort known to us is the introduction before 1879 of the helicid snail *Cornu aspersum* (formerly *Helix aspersa*) to South Australia in the hope of eradicating alien slug species. *Cornu aspersum* is itself now a significant agricultural and garden pest in Australia (e.g., [21,22]).

Here, we first provide a brief historical overview of two infamous instances of non-target impacts of biocontrol programs on non-marine molluscs using snails of the genus *Euglandina* and the flatworm *Platydemus manokwari*. We then review three less well-known biocontrol programs targeting pest non-marine molluscs with the aim of identifying potential threats to non-target species. These programs include the use of sciomyzid flies to control the lymnaeid intermediate host of the liver fluke *Fasciola gigantica*, and sarcophagid flies and the nematode *Phasmarhabditis hermaphrodita* to control pest terrestrial molluscs. Finally, we highlight two contemporary programs to illustrate how they fail to meet the standards for current best practices for ensuring the environmental safety of proposed introductions of alien biocontrol agents.

## 2. Historical Review: Biocontrol of Non-Marine Molluscs in Oceania

The sad story of non-target impacts of previous biocontrol programs using the snails *Euglandina* spp. and the flatworm *Platydemus manokwari* targeting the land snail pest *Lissachatina fulica* has been recently reviewed in detail by Gerlach et al. [20]. Therefore, we will provide just a brief overview of these two failed biocontrol programs and include a lesser-known historical biocontrol introduction to establish the context for our critical view of using generalist biocontrol agents to control non-marine molluscs.

### 2.1. Euglandina “rosea” Species Complex

In 1936, the giant African land snail, *Lissachatina fulica*, became established in the Hawaiian Islands and, subsequently, on various islands in Micronesia [23,24,25,26]. By the mid-1940s, it was perceived as a significant agricultural pest and, beginning in 1947, the Pacific Science Board of the U.S. National Research Council, the Board of Agriculture and Forestry of the Territory of Hawaii, and the Hawaiian Sugar Planters Association conducted a search for potential biological control agents [24,27]. Seventeen species of predatory snails were released in the Hawaiian Islands for the control of *L. fulica* and other species. Of those released, at least three, *Gonaxis kibweziensis*, *G. quadrilateralis*, and *Euglandina* “*rosea*”, have become established [28,29]. These snails, especially *Euglandina* “*rosea*”, have subsequently been introduced to numerous Pacific islands and beyond as part of efforts to control *L. fulica* and other snail pests [20,26].

It is now known that at least two species of *Euglandina* have been imported into the Pacific under the name *E.* “*rosea*” [19]. For this reason, from here on we generally refer to these two species collectively as *Euglandina* spp., as in few cases is it known which species was introduced or even if the introduction was restricted to only these two species.

From the start, malacologists protested the proposed introduction of these and other predatory species and predicted that doing so would mean the probable destruction of native land snail faunas while failing to control *L. fulica* [30,31,32]. Despite the lack of evidence for effectiveness of this control agent, agricultural authorities continued to introduce *Euglandina* spp. to additional island groups into the 1990s [20]. While failing to control *L. fulica* [7,8,12,17,33,34,35], *Euglandina* spp. have been recorded as probably the main cause of numerous endemic land snail extinctions, especially in the Hawaiian Islands and French Polynesia [11,12,33,36,37,38,39,40,41,42,43,44,45] as well as Bermuda [46,47,48,49] and the islands of Mauritius and Rodrigues in the Indian Ocean [50].

### 2.2. Platydemus manokwari

The introduction of *Platydemus manokwari* has been credited with substantially reducing *L. fulica* populations in the Philippines [51], Maldive Islands [52,53], New Guinea, Guam, and Samoa [54,55]. However, the perceived control of *L. fulica*, if true, came with numerous non-target casualties, as *P. manokwari* is a highly invasive species and has become a serious threat to the native snail faunas of the Mariana Islands, Samoa (formerly Western Samoa), and the Ogasawara Islands of Japan [17,56]. In the Ogasawara Islands, the extinction of most of the endemic *Mandarina* species, and others, of the main island of Chichijima was due to predation by *P. manokwari* [17,45,57,58,59,60,61,62,63]. Ironically, even the introduced *Euglandina* spp. are now almost extinct on Chichijima because of *P. manokwari* [56]. Since the first record of *P. manokwari* in Florida in 2012 [64], it has spread widely within the state [65] and some surveyed populations of the iconic tree snails, *Liguus* spp. and *Orthalicus* spp., are suffering from intense predation, and their numbers have declined drastically ([20], T. M. Collins, pers. comm. 2020).

*Platydemus manokwari* is present on many other islands and archipelagos in the Indo-Pacific, as well as in Puerto Rico, Florida, Australia, France, Thailand, Hong Kong, and Guadeloupe (see references in [17,64,66,67,68]). With notable exceptions, most of these introductions of *P. manokwari* were probably accidental, but clearly *P. manokwari* is a voracious polyphagous species [63] and substantial non-target impacts are to be expected from its introduction. The reports of successful biocontrol by *P. manokwari* are anecdotal accounts unsupported by rigorous pre- and post-release surveys of *L. fulica* populations or any consideration of possible impacts on non-target species. Whether or not it is an efficient biocontrol agent, however, it is well-established that its introduction can have devastating impacts on the native land snails of the islands on which it becomes established. Claims of successful control of *L. fulica* by *P. manokwari* are unverified.

### 2.3. Sciomyzid Flies

Nearly all flies of the family Sciomyzidae are saprophages, parasitoids, and/or predators of non-marine molluscs. Most target freshwater snails, while some target terrestrial ones; a few target freshwater bivalves or other invertebrates [69]. Because of their snail-killing abilities, several species have been investigated as potential agents for the biological control of pest non-marine molluscs [69,70,71]. Their potential use as biological control agents was first suggested by Berg [72], and subsequently considerable attention focused on their use in the control of aquatic snails that are intermediate hosts of the trematode worms causing schistosomiasis, an important human disease in Asia, Africa, and the Neotropics (e.g., [70,73]).

Beginning in 1958, eleven sciomyzid species were released in the Hawaiian Islands for control of the aquatic lymnaeid snails that are the principal intermediate hosts in Hawaii of the liver fluke *Fasciola gigantica*, the causative agent of fascioliasis in cattle and, occasionally, humans [74]. Two of these sciomyzid flies, *Sepedomerus macropus* and *Sepedon aenescens*, have become established in Hawaii [28,69]. *Orientogalba viridis*, one of the alien lymnaeids established in Hawaii, is also present on Guam, where it is the host of the liver fluke *Fasciola hepatica* [75]. Two of the species released in Hawaii, *Sepedomerus* *macropus* and *Sciomyza dorsta*, along with 106 other insects, were released for biocontrol on Guam in 1959 and 1961 [76], and at least one of the sciomyzids, *S. macropus*, is established there [71].

Hawaii has no native sciomyzids [77,78], so the introduction of novel snail-killing agents raised the question of possible effects on non-target species. In general, sciomyzids are not narrowly host-specific, a circumstance that further raises such concerns [71], and the lack of host specificity of one of the established sciomyzids, *S. aenescens*, has been demonstrated in Hawaii by its association with an invasive terrestrial snail, *Oxychilus alliarius* [78]. Van der Schalie [32] flagged the threat to Hawaii’s native lymnaeids, and Knutson and Vala [69] confirmed that no pre-release studies had been done on the potential effects on native lymnaeids. They also noted that van der Schalie’s fear that the flies would spread beyond the points of their release had been borne out and agreed that it was likely that these alien sciomyzids would attack any lymnaeid, including native species.

At least four endemic lymnaeid species inhabit the Hawaiian Islands [79]. One of the endemic species cited by van der Schalie [32], *Erinna newcombi*, also known as Newcomb’s snail, has been listed by the U.S. Fish and Wildlife Service (USFWS) under the U.S. Endangered Species Act [80]. When listing it, the USFWS [80] cited the two biocontrol sciomyzid fly species as significant threats to the survival of Newcomb’s snail and other native lymnaeid snails, noting that these flies had extended their range into areas inhabited by *E. newcombi*. With the lack of pre- and post-introduction studies on the population distribution and size of the non-native lymnaeids, it is impossible to assess the effectiveness of sciomyzids as biocontrol agents [32,71]. However, two non-native lymnaeid species, *O. viridis* and *Pseudosuccinea columella*, are still present in Hawaii. Given that the introduction of sciomyzid flies may severely impact native non-marine snails, they should not be considered as snail biocontrol agents.

### 2.4. Molluscan Biocontrol in Oceania: A Summary

The USFWS has listed nine species and one entire genus of land snails from U.S. islands in the Pacific as “endangered” or “threatened” under the Endangered Species Act. The Service has determined that the survival of every one of them is at risk because of biological control agents intentionally introduced by agricultural authorities [80,81,82,83,84]. As noted above, the survival of *Erinna newcombi* is threatened by sciomyzid flies, but also by *Euglandina* spp. [80,85]. Additionally, it has not been shown that these biological control introductions are effective agents for the control of their intended target species: *L. fulica* [8,12] and non-native lymnaeids [32,71]. Thus, the balancing of economic benefits versus environmental harm weighs heavily against their use. For these reasons, these species should not be considered as snail biocontrol agents. Given the horrible track record of molluscan biocontrol efforts in the islands of the Pacific, proposals for future such efforts must be given the most careful scrutiny.

### 2.5. We Don’t Do That Any More

Skeptics of proposed anti-mollusc biocontrol proposals who cite the use of *Euglandina* spp. and other ecological biocontrol disasters as a reason for caution in molluscan biocontrol are routinely met with the response that can be paraphrased as “those were the bad old days; we don’t do that anymore”. It is claimed that modern biocontrol projects should not have to bear the taint of early biocontrol efforts as the disasters of these indiscriminate introductions were “carried out by untrained individuals with no state supervision” [86] (p. 95) and “would never pass biocontrol scrutiny today” [87] (p. 18). These failed biocontrol efforts include the introduction of the small Indian mongoose (*Herpestes aureopunctata*), the Common Mynah (*Acridotheres tristis*) and cane toads (*Rhinella marina*; [28,87,88,89,90]).

We do not address possible hazards associated with these biocontrol programs. Our concern is primarily with molluscan biocontrol, although many of the issues we discuss are equally applicable across all biocontrol programs. Where molluscan biocontrol is concerned, the papers cited above seriously misrepresent the record. The biological control agents introduced to Hawaii and the intentional distribution of *Euglandina* spp. to numerous other islands in the Pacific were not done by “untrained individuals with no state supervision” [86] (p. 95). Instead, they were done by “trained entomologists” [86] (p. 95)—but not trained malacologists—acting under the authority of the Territory and subsequently State of Hawaii in the 1950s through 1980s, and often over the timely objections of malacologists and others [30,31,32,91]. Additionally, Hawaii’s regulatory system was established in 1944, and Lai [92] and Funasaki et al. [28] touted this regulatory scheme as highly effective in ensuring that the released biocontrol agents did not pose harm to human health or native ecosystems; nevertheless, this regulatory scheme failed to prevent the introduction to Hawaii of *Euglandina* spp. or the sciomyzids.

Snails are not insects, and all too often agricultural authorities have failed to recognize that critical distinction. None of the agents used to date in the Pacific for molluscan biocontrol are narrowly target-specific. Instead, they are generalist predators, exactly the sort of agents modern standards reject. In light of this history, malacologists are rightly skeptical of biocontrol proposals targeting non-marine molluscs.

## 3. Current Best Practices, Sarcophagid Flies, and *Phasmarhabditis*

Have we moved away from the “bad old days” and toward the use only of narrowly target-specific agents for the biocontrol of molluscan pests? Given that: (1) only around 1.9 million species of organisms are known to taxonomists [93] of the estimated 8–9 million or more on Earth [94], and (2) the conservation status of only 8.5–10% of all molluscan taxa known has been evaluated, with over one third of those evaluated being poorly known (categorized as “Data Deficient”; [44]), any affirmative answer to this question is dubious at best, simply because the potential vulnerability and distributions of so many species that may be impacted remains unknown. To further illustrate concerning issues with biocontrol as it is applied to molluscs, we outline current best practices in biocontrol safety and will evaluate those standards using two agents currently in use in Australia and Europe, sarcophagid flies and the nematode *Phasmarhabditis hermaphrodita*, to see whether they meet that test.

### 3.1. Best Practices

Various national, regional, and international agencies have developed guidelines and agreements for the release of biological control agents (at least for weeds), briefly reviewed by Babendreier [90]. A key international document is the FAO *Guidelines for the Export, Shipment, Import and Release of Biological Control Agents and Other Beneficial Organisms*, originally adopted in 1995 and published in 1996, revised in 2005, and further revised in 2017 [95]. However, these guidelines focus on phytosanitary issues, and when mentioning non-target impacts, seem to do so as something of an afterthought. Balciunas [96,97] developed an accessible, short, 12 bullet-point international *Code of Best Practices for Classical Biological Control of Weeds* (Table 1), which has been adopted by the USFWS [98] and is equally applicable to control of pest animals. We consider this *Code* to be too general and not focused specifically on non-target impacts or potential host ranges of biological control agents. There is no equivalent code for the use of predators, parasites, or pathogens to control animal pests. However, the multi-authored book edited by Van Driesche and Reardon [99] goes a long way to filling that gap, specifically regarding the non-target effects of such biocontrol agents, with the introductory and concluding chapters addressing key issues and problems and possible ways of addressing them. Of course, it only addresses the control of pest arthropods, and may also be somewhat out of date.

### 3.2. Sarcophagid Flies

Some members of the dipteran family Sarcophagidae (flesh flies) are parasitoids of terrestrial molluscs and accordingly have attracted attention as potential biocontrol agents, especially in Australia where alien helicoid snails have become significant agricultural pests [101,102,103]. Research has focused on species of *Sarcophaga* native to the Mediterranean region where the pest snails are also native. A species found in France and first identified as *Sarcophaga penicillata* was determined to be sufficiently host-specific to allow its use, and the flies were released in South Australia in 2000 and thereafter [104,105]. By 2007, the fly had become established in the release area “in low numbers” [105] (p. 11). Subsequently, it was determined that the species initially identified as *S. penicillata* was in fact *S. villeneuveana* [106,107]. Two other species of *Sarcophaga* were rejected as being insufficiently host specific [105]. Nearly two decades later, it was determined that biocontrol using French *S. villeneuveana* had failed [107]. Genetic studies of Australian populations of *C. acuta*, were undertaken, and it was determined that the Australian snails were most closely related to source populations in Morocco and the Iberian Peninsula, not France [106], and studies were begun to determine whether *S. villeneuveana* from the Iberian Peninsula or Morocco might be more effective in controlling *C. acuta* [106,107,108], and Moroccan *S. villeneuveana* have been brought to Australia for further testing and potential release [109]. The initial uncertainty as to the identity of the sarcophagids under study highlights the fact that, as was the case with *Euglandina* spp., even modern biological controls that are touted as not being ‘classic biocontrol’ are still based on inadequate evaluation of the biology and even identity of appropriate predators and parasitoids. Despite more than a century of studies exploring the complexity of natural selection, population variability, and evolutionary adaptation, biocontrol advocates still fail to consider the role of such complex evolutionary and ecological processes in evaluating potential impacts, or factor in the complexity of the systems with which they are working.

The Australian *Sarcophaga* program has been praised for its attention to non-target impacts and its rejection of prospective biocontrol agents found not to be host-specific [7]. Nevertheless, some concerns remain about the use of *Sarcophaga* spp. in Australia and their effects on non-target terrestrial molluscs. Despite following Australian quarantine protocols, it could not be concluded that *S. villeneuveana* was host specific. First, although the initial vetting process has not been described in detail, it is clear that *S. villeneuveana* is not narrowly host-specific, as one (unnamed) native species (of 36 tested) was found to be “possibly attacked” by it [7] (p. 136). Second, although it has been almost two decades since *S. villeneuveana* was first released in Australia, there is nothing in the literature to suggest that field studies have been undertaken to determine whether or not native species are in fact being affected, or whether the fly has spread beyond the release sites into nearby areas inhabited by native species. Finally, as there appears to have been no report that the program has provided any economic benefit in terms of reduced pest populations, the balance between the uncertain risks and the so-far apparent lack of benefits would seem to weigh against further releases in the absence of strong evidence that Iberian flies will perform substantially better than their French conspecifics, while also not impacting native species.

### 3.3. Phasmarhabditis spp.

*Phasmarhabditis hermaphrodita* (Nematoda: Rhabditidae) is a parasitic nematode lethal to many terrestrial molluscs and used as a biological control agent for pest molluscs [110,111]. It occurs in Europe, Iran, Chile, Egypt, and New Zealand [112,113,114] and has recently been found in the United States [111,115,116]. Its life history is complex, as is the manner in which it and its associated bacterium, *Moraxella osloensis*, infect and kill host molluscs [112,117] in a manner analogous to that of entomopathogenic nematodes (EPN), which with their associated bacteria kill insects, and which are also available as commercial pesticides (e.g., [118]). There is evidence, however, that the association with *M. osloensis* is merely facultative, as *M. osloensis* was not found associated with recently discovered populations of *P. hermaphrodita* in Oregon [119]. In light of the difference in its choice of target organisms, *P. hermaphrodita* is not in fact an EPN, but will be referred to here as a malacopathogenic nematode (MPN), following McDonnell et al. [120].

Preparations containing *P. hermaphrodita* are available for sale as a biocontrol agent in Europe [121], Canada [122], and Kenya [123]. It is not yet available in the United States, although proposals for its use here are likely [118]. Laboratory tests of three *Phasmarhabditis* species, including *P. hermaphrodita*, have been undertaken in the United States, demonstrating their efficacy against *Theba pisana*, a significant pest in California [124]. Notwithstanding legal prohibitions on its importation, however, genetic analysis of samples of *P. hermaphrodita* from North America (California and Oregon) and New Zealand indicated that they were probably derived from unauthorized distribution of commercial preparations of *Phasmarhabditis* spp. [119].

It has also been suggested that *P. hermaphrodita* might be used in tandem with the sciomyzid fly *Tetanocera elata* (Fabricius) in an integrated pest management program targeting pest terrestrial molluscs, but the effectiveness of this approach thus far has not been supported [125]. Potential hazards of the use of sciomyzid flies in molluscan biocontrol are discussed above.

It is claimed that the use of *Phasmarhabditis hermaphrodita* as a biocontrol agent poses risks to non-target molluscs that are at most minimal (e.g., [117,126,127]), and that it “is relatively safe to non-target species” [128] (p. 1483). Although it is said that *P. hermaphrodita* and EPNs have a “wide host range” [129] (p. 347), they are nevertheless claimed to be “safe for humans, other non-target organisms and virtually posing no hazardous effect on the environment” [129] (p. 348). Although “its affect on non-target organisms has not been extensively studied”, we have been assured that “the information available makes the evidence clear that [*P. hermaphrodita*] is safe for non-target snails” [129] (p. 368). Given the limited information available on this topic, skepticism is warranted.

Notwithstanding the paucity of studies of its effects on non-target species, we do know that in addition to species of the families Limacidae, Arionidae, Milacidae, and Veronicellidae, several helicid species are susceptible to *P. hermaphrodita* [111,112,130], as are the achatinid *Lissachatina fulica* [120] and an athoracophorid species endemic to New Zealand [114]. There are also contradictory reports of susceptibility of the freshwater snail *Lymnaea stagnalis* [111,131]. The legally protected species *Geomalacus maculosus*, on the other hand, has been found not to be attacked by *P. hermaphrodita* [132]. It appears, then, that not all land and freshwater snail species are equally susceptible to *P. hermaphrodita*. Nevertheless, the broad taxonomic range, spanning two gastropod orders (Stylommatophora and Systellommatophora), of those species known to be vulnerable to it raises legitimate doubts as to its supposed benign nature.

Furthermore, at least one of the arguments for its lack of impact on non-target snails is based on a faulty assumption. Wilson and Grewal [133] (p. 427) admitted that “[l]ittle work has been done on the effects of *P. hermaphrodita* on non-target organisms.” They nevertheless concluded that “[i]t is unlikely that many snail species would come into contact with *P. hermaphrodita* as they tend to live in plants above the ground, unlike the shell-less slugs that live in the soil (Mengert 1953). Thus, the threat to non-target snails is unlikely to be high.” Additionally, Wilson et al. [127] (p. 716), asserted that “non-target snails, unlike slugs, tend to live on sites above the soil eg [*sic*] on plant foliage, stems and stones, and thus out of contact with soil dwelling nematodes”. The highly erroneous assumption that ground-dwelling snails are few indicates a troubling lack of knowledge of land snail biology on the part of some proponents of the use of *P. hermaphrodita*. In actuality, such snails play a major role in decomposition of leaf litter and in soil formation [134,135,136] and are undoubtedly more numerous than tree-dwelling species in most habitats globally (e.g., [137,138]), including in locations well known for their arboreal species (e.g., [139]). Indeed, freshwater species may also be at risk. As “[t]he complete host range of *P. hermaphrodita* is poorly understood and many slug and snail species have never been tested for their susceptibility toward this nematode” [131] (p. 679), it would be unreasonable to assume the absence of adverse impacts on non-target species.

*Phasmarhabditis hermaphrodita* has only recently been reported from the United States [111,116] and it cannot be presumed to be indigenous here as in Britain [127]. Thus, its ecological impact may be greater than in regions where it has been long established. Accordingly, there is much we do not know regarding potential non-target impacts of the use of *P. hermaphrodita* as a biocontrol agent. A more thorough understanding of the biology and host range of candidate *Phasmarhabditis* spp., comprehensive host range testing that incorporates native gastropod species, and input from expert malacologists are essential in assessing the potential safety and effective use of *Phasmarhabditis* spp. as pest control agents in the U.S. [140]. Additionally, alternative management practices with lower potential for negative impacts should be explored. At least one study has shown that the application of *P. hermaphrodita* is not as effective at keeping slug numbers low as simple management practices, such as grass mowing [141]. This study echoes our sentiments in emphasizing how a little understanding of basic biology can go a long way to developing effective controls that do not further endanger other species.

### 3.4. Moraxella osloensis as a Human Pathogen

An additional hazard not presented by the other biocontrol agents discussed here but that must be considered in assessing risks of use of the *P. hermaphrodita/M. osloensis* partnership in biological control is that *M. osloensis* is a human pathogen. Unlike EPNs already approved for use in the United States, it is neither dependent on a host nematode nor innocuous to humans. Instead, it is a component of the flora of the nasal passages of humans [142], and a preliminary literature search for reports of *M. osloensis* as a cause of human disease showed that it can be a causative agent for a broad range of conditions, including peritonitis [143,144], endocarditis [145,146], meningitis [147,148], osteomyelitis [149,150], eye infections [151,152], and others [153]. Though such infections are uncommon, Graham et al. [154] reported that from 1953 to 1980, *M. osloensis* had been recovered from 199 samples submitted to the U.S. Centers for Disease Control and Prevention for analysis.

Uncertainties regarding impacts on non-target mollusc species and health risks possibly associated with dissemination of *M. osloensis* must be considered in regulatory decisions regarding potential use of *P. hermaphrodita* in the United States. Formerly, legislation prohibited the sale of *P. hermaphrodita* in the United States as it was not known to occur here [112]. Its recent discovery in California and Oregon [111,115,116] changes the regulatory framework, but various federal and state approvals would nevertheless be required. Certain EPNs are considered to pose little threat to non-target organisms or the environment and thus are not subject to federal pesticide registration requirements [118], and some EPNs and their associated bacteria have been exempted by the U.S. Environmental Protection Agency (EPA) from regulation under the Federal Insecticide, Fungicide, and Rodenticide Act (FIFRA) [155,156]. They are instead regulated by the United States Department of Agriculture [157]. While this may be appropriate for EPNs targeting insects, the paucity of information on the effects of *P. hermaphrodita* on non-target molluscs should preclude it and other *Phasmarhabditis* spp. from being treated similarly at this time.

As noted above, it has been assumed that the use of EPNs is environmentally safe [158,159]. In 1987, the EPA “determined that all strains and species of insect-parasite nematodes of the genera *Steinernema* and *Heterorhabditis* with their associated bacteria *Xenorhabdus nematophilus* (Thomas and Poinar) and *X. luminescens* (Thomas and Poinar) are exempted from registration under [FIFRA]” because “[t]hese bacteria are strongly host dependent and are not known to survive for long periods outside their nematode symbionts or secondary host insects [and therefore] it is unlikely they could or would cause infection in mammals” [155] (p. 54). Instead, these organisms are regulated by the U.S. Department of Agriculture under the authority of the Plant Protection Act, 7 U.S.C. §§ 7701 et seq. “As a biocontrol agent they [EPN] offer an ecologically safe alternative to chemical pesticides. Therefore, the EPA has exempted them from all registration requirement and related regulation” [160] (p. 2). It cannot be assumed that a similar exemption would be applicable to *Phasmarhabditis* spp. Unlike *Xenorhabdus* spp., *M. osloensis* is not host dependent and does cause infections in mammals, including humans. As the factual predicate for exemption of the *Xenorhabdus* species does not exist for *M. osloensis,* it is doubtful that *P. hermaphrodita* would be eligible for an exemption from regulation under FIFRA of the kind that was granted to *Steinernema* spp. and *Heterorhabditis* spp., at least if *M. osloensis* is included in the preparation. It has been suggested that *P. hermaphrodita* may be pathogenic to molluscs even in the absence of *M. osloensis* [161,162]. If that is so, preparations of *P. hermaphrodita* that do not include *Moraxella* or other associated bacteria would not raise the question of the applicability of FIFRA. A recent study [119] found that some North American populations of *P. hermaphrodita* are not associated with *M. osloensis*, a circumstance that would have implications for the applicability of FIFRA. Concerns regarding impacts on non-target mollusc species would of course remain to be resolved and require sufficient study to reliably evaluate such impacts.

## 4. Defining Success in Modern Biocontrol

Contemporary biocontrol is often touted as successful and accompanied by claims that it is carried out in a rigorously controlled regulatory framework that has benefited from more than a century of lessons learned from the days when “nobody really cared about risk” [87] (p. 18). Although we agree that the regulatory process for approval of biocontrol agents has dramatically improved, and additional, although still inadequate, studies are carried out to better evaluate impacts on non-targets pre-release, there remain enormous gaps in understanding of taxonomy, ecology, and the myriad complex biological interactions of introduced species in novel environments. Few studies produce adequate evidence to draw the often-touted conclusion that biocontrol is safe and successful [163], typically narrowly defining success as a reduction in the population size of single target species and a few closely related non-targets. There are rarely any efforts to evaluate other potential non-targets, indirect effects, or to assess ecosystem level parameters that are important for the long term success of species. Furthermore, most studies are poorly designed to effectively evaluate the potential non-target impacts of biocontrol agents pre- or post-release, and the fact that only around 25% of the estimated 8 million or more species of multicellular plants and animals on Earth have been described, makes such claims even less credible. How is it possible to evaluate impacts of a biocontrol vector on non-targets when only a quarter of the diversity in a given ecosystem is described? The same taxonomic impediments [164] that impede efforts to document and conserve biodiversity are also at play when attempting to evaluate potential impacts from introduced biocontrol agents. These include a lack of well-trained taxonomists in most groups of invertebrates, particularly hyperdiverse groups like insects and molluscs [165].

Studies aimed at developing and evaluating biocontrols that do not account for such gaps in knowledge are at best data deficient. The onus needs to be on those wishing to release these biocontrol agents in the midst of a biodiversity crisis (e.g., [166]) to more rigorously design and implement studies both pre- and post-release that demonstrate not only successful reduction of the target species, but also negligible impacts on non-targets across the ecosystem. Failure to do so negates any claims of modern biocontrol being safe, effective, and successful, regardless of how well it controls the target. The costs are just too high! Proponents of new molluscan biocontrol programs must ensure that they are not creating the new rosy wolfsnail or New Guinea flatworm [20].

Many of the recommendations that we could offer are already part of many biocontrol plans, but are rarely fully or effectively implemented (see [167]). These and other recommendations for defining success and developing effective biocontrols specifically for molluscs include (1) an adequate understanding of the systematics of both the biocontrol agent and the potentials targets, (2) a pre-release inventory of all native species that could be potentially impacted by the biocontrol agent, (3) assessment and screening of potential pathogens and parasites that might be carried by the proposed biocontrol agent, (4) inclusion of controls for evaluating declines in target species, (5) follow up surveys to evaluate both target and non-targets, and (6) integration of malacologists knowledgeable in the biology of both pest and predator, and in the basic biology and conservation aspects of non-target species in the development and assessment of biocontrol programs. While this list is not comprehensive, the effective implementation of these recommendations would go a long way to addressing many of the problems that currently make the costs of molluscan biocontrol programs too high.

## 5. Conclusions

Sixty-five years ago, the malacologist (and trained entomologist) Albert R. Mead [31] warned that snails are not insects and that biocontrol programs targeting them need to take that distinction fully into account.

The stepping off point in the problem seems to have been the application of the principles of insect biological control to the then new field of molluscan biological control. The citing of years of experience in the field of insect biological control as appropriate to the control of mollusc pests, and thus as ipso facto qualification for taking irreversible steps in a wholly different and biologically little-known group of animals, was and remains unconvincing. We submit that no one can safely reason a priori from the results of experiments in one phyletic group to comparatively similar experiments in another, especially in an area of biology that is as treacherous as it has proven itself to be so often in the past.

Molluscan biocontrol is no longer a new field, but the intervening years have only demonstrated that the concerns of Mead, Fosberg, and van der Schalie were, if anything, understated. We submit that scientists whose major focus is the study of the biology of non-marine molluscs, not entomologists or others whose primary interest is in the control and eradication of non-marine molluscs, have been and continue to be virtually unanimous in being highly skeptical of the safety of programs of biological control targeting these animals. Proponents of the molluscan biocontrol programs discussed above have not generally involved malacologists with an interest in biodiversity conservation in the planning or evaluation of the efficacy and safety of these programs. To the extent current standards for evaluating the efficacy and safety of such programs are inadequate, McDonnell et al. (2020 [168]) have noted that we have many knowledge gaps to fill and acknowledge that an essential part of the solution to filling these gaps needs to include the engagement of gastropod biologists actively involved in conservation. We urge those planning such programs to involve malacologists at an early stage to ensure the participation of specialists knowledgeable in the biology of these molluscs.

We do not claim that no anti-molluscan biocontrol programs have been successful, or that the cost/benefit analysis could never tip in favor of going forward with such a program. Use of the thiarids *Melanoides tuberculata* and *Tarebia granifera*, as well as other aquatic molluscs as competitors of the intermediate hosts of schistosome blood flukes, has shown some effectiveness (e.g., [168,169,170]), although the causal mechanism of competition is in some cases unknown [12]. The human costs of schistosomiasis are huge and must be taken into account; even so, such programs are not without environmental hazards. Both *M. tuberculata* and *T. granifera* are themselves highly invasive species that can displace native species [168,171,172] and that host their own suites of trematode parasites, eleven of which, in the case of *M. tuberculata*, may infect humans, including the liver fluke ***Opisthorchis*** *sinensis* and the lung fluke *Paragonimus westermani* [173]. Furthermore, introductions of these species have caused the disappearance of some native species [168]. In addition to calling for more rigorous studies of potential pre-and post-release impacts, we also ask that the benefits of a particular biocontrol agent not be overstated through unverified claims of effectiveness or that the costs be understated through unwarranted claims of an absence of non-target impacts without rigorously evaluated, empirically derived data to support such claims. Finally, we urge those advocating biocontrol to also evaluate alternative management practices, many of which may prove as effective and less environmentally costly.

## Figures and Tables

**Table 1:** The International Code of Best Practices for Classical Biological Control of Weeds [96,97,100].

1. Ensure target weed’s potential impact justifies release of non-endemic agents
2. Obtain multi-agency approval for target
3. Select agents with potential to control target
4. Release safe and approved agents
5. Ensure only the intended agent is released
6. Use appropriate protocols for release and documentation
7. Monitor impact on target
8. Stop releases of ineffective agents, or when control is achieved
9. Monitor impacts on potential non-targets
10. Encourage assessment of changes in plant and animal communities
11. Monitor interaction among agents
12. Communicate results to public

## Data Availability

Not applicable.
